# Determinants of capital structure: a case of hospitals in China

**DOI:** 10.1186/s12913-025-13263-x

**Published:** 2025-08-15

**Authors:** Qi Zhang, Audrey Laporte

**Affiliations:** 1 Institute of Health Policy, Management and Evaluation, University of Toronto, Toronto, Canada; 2 Canadian Centre for Health Economics, Toronto, Canada

**Keywords:** Hospital, Capital structure, Asset, Debt, China

## Abstract

**Background:**

Hospitals possess distinct characteristics that influence their capital structures, particularly in China, where the hospital industry is predominantly public but includes private not-for-profit and private for-profit hospitals. There is currently limited empirical evidence on how operational and financial factors influence the debt-to-asset decisions of hospitals in China.

**Methods:**

This study analyzed data from 909 hospitals in China collected in 2013 and 2014 through the China National Health Statistical Information Report. Using a two-part model, we examined the effects of ownership, hospital type, revenue stream diversification, market share, and other characteristics on short-term, long-term, and total debt-to-asset ratios.

**Results:**

The analysis revealed that private for-profit (FP) hospitals and specialized hospitals did not significantly reduce the probability of assuming short-term debt. However, private FP hospitals with less diversified revenue streams were more likely to rely on short-term debt to meet operational needs. Private ownership also reduced the probability of assuming long-term debt. Higher returns on assets (ROA) were significantly associated with a lower probability of assuming any debt or short-term debt but had no significant effect on the likelihood of assuming long-term debt. Most hospital characteristics were not significant predictors of short-term or long-term debt-to-asset ratios, with the exception that being a general hospital was linked to higher long-term debt-to-asset ratios. Notably, private FP hospitals with higher ROA were associated with increased long-term debt-to-asset ratios, indicating that profitability plays a key role in their long-term borrowing decisions.

**Conclusion:**

This study highlights the influence of financial and operational factors on hospital capital structures in China. The results emphasize the need for policies to support private hospitals, particularly in obtaining long-term loans and diversifying financing options, to promote equitable access to funding and improve healthcare delivery. These insights contribute to the understanding of hospital debt dynamics and provide implications for healthcare policy and management.

## Background

The hospital sector contains three marked features that have potential implications for its capital structure. First, the majority of of hospitals are nonprofit organizations, either public hospitals operated by the government or organized as private not-for-profit (NFP) institutions. In China, the hospital industry is dominated by public hospitals [1]. However, there is an increasing number of private NFP and for-profit (FP) hospitals since the health system reforms initiated in 2009. Hospitals face a financing predicament despite its ownership. Too little leverage fail to provide enough resources for growth, while too much may lead to insolvency or even bankruptcy. For instance, there is evidence suggest that found that excess short-term loans may be the most important cause for hospital bankruptcy in South Korea [2].

A second feature of the sector is that hospitals are typically categorized based on their ability to admit and provide care for patients. For instance, there are general hospitals, specialized hospitals, and Traditional Chinese Medicine hospitals. The type of hospital affects the operation characteristics of the hospital, which in turn influences the relative levels of debt and equity in the hospital. In China, there is a categorization system in which hospitals are categorized into three different levels based on their functions, tasks, infrastructure, technology, medical services provided and management. According to the National Health Commission of China, the basic conditions and requirements of each level are reported in Table 1 [3]. Generally, higher level hospitals set higher prices for medical services, are more likely to be public hospitals and are in priority to receive government subsidies [3].

**Table 1:** Main features of the hospital classification system in China

Levels^a^	Beds	staff	Coverage	Function
Level 1	20-99	$\ge$ 0.7 doctors per bed	Township	Provide fundamental medical services
		$\ge$ 3 doctors, $\ge$ 5 nurses	Community	
Level 2	100-499	$\ge$ 0.88 doctors per bed	Region	Provide comprehensive medical services
		$\ge$ 0.4 nurses per bed	County	Take some research and teaching task
Level 3	$\ge$ 500	$\ge$ 1.04 doctors per bed	Municipality	Provide specialist medical services
		$\ge$ 0.4 nurses per bed	Province/Nation	Solve intractable/incurable diseases
				Take higher education and research task

^a^There are also unclassified hospitals. These hospitals may fail to meet the requirement for level 1 or have a undergoing rating process

While hospitals primarily generate revenue by providing medical services, they also have other revenues sources such as education programs and government subsidies. Some studies suggest that a more diversified revenue structure increases the long-term liability capacity among art and culture nonprofit organizations [4, 5]. However, little has been revealed about whether and how the revenue diversification influences a hospital’s borrowing behavior concerning both short-term and long-term debt.

The main purpose of this study is to examine the factors shaping the capital structure of a hospital. Using hospital-level financial data from the China National Health Statistical Information Report, we propose and test a two-part model of hospital borrowing behavior. This cross-sectional analysis assesses the effect of ownership, hospital type, revenue stream, and other hospital characteristics on the hospital total debt-to-asset ratios as well as short-term and long-term debt-to-asset ratios. To our knowledge, this study represents the inaugural exploration into the determinants of leverage decisions among hospitals in China. The insights gleaned from our research not only augment the limited literature on hospital capital structure but also offer novel perspectives on the debt financing decision-making processes within the Chinese context.

In the course of our analysis, we address another issue of testing two competing finance theories on capital structure. The static trade-off theory argues that hospitals balance the costs and benefits of debt to decide how much to borrow. In contrast, the pecking order theory suggests that hospitals prefer using internal funds before seeking loans. Our findings support the pecking order theory for short-term borrowing, while for-profit hospitals seem to follow the trade-off theory for long-term loans.

The rest of the paper proceeds as follows: A brief background of hospitals’ financing strategies in China is outlined. The next section discusses the two dominant capital structure theories and empirical findings in the capital structure literature. Then, data, variable definitions, and empirical strategy are described. The results are presented, followed by a discussion and conclusions.

### Public hospitals in China’s healthcare financing reforms

The escalation of total health expenditure is a prevailing issue across many nations [6, 7]. In China, total health expenditure has expanded significantly since the inception of economic reforms in 1978, consistently outpacing GDP growth [8]. To mitigate this growth, China initiated comprehensive healthcare reforms in 2009, aiming to deliver affordable and equitable healthcare [9].

Historically, public hospitals in China have primarily relied on revenue from service charges based on a fee-for-service model, profit margins from drug sales, and government budgetary allocations [1]. Additionally, the government has regulated prices for pharmaceuticals and medical services offered by public hospitals. While service charges for basic medical services like surgery, diagnosis, therapy, and nursing have been modest, they have been highly lucrative for services involving advanced technologies such as computed tomography and magnetic resonance imaging [10].

The financing reform in China targeted four key areas: eliminating drug mark-ups, increasing budget allocations, revising fee schedules, and reforming payment mechanisms. The Chinese government phased out drug mark-ups in public hospitals, excluding traditional herbal medicines, culminating in their complete elimination by 2018. Additionally, a goal was established to reduce the share of pharmaceutical revenue to 30% of total hospital income. To compensate for the revenue shortfall from the elimination of mark-ups, price hikes for medical services were projected to account for 40-90% of the lost revenue, varying by province. Government subsidies were expected to cover an additional 10-50%, while hospitals would absorb the remaining costs through efficiency improvements in management.

The impact of health financing reforms on the financial situation of public hospitals remains ambiguous. For instance, the allocation of government subsidies is contingent upon municipal or county governments responsible for the hospitals, leading to considerable variation based on local fiscal conditions. Additionally, some local governments are beginning to adopt payment models for hospitals, such as capitated global budgets or diagnosis-related groups. These reforms are too recent to fully assess their effects on hospital capital structure. However, research indicates that these reforms have introduced new challenges, including insufficient medical insurance funding, inconsistent insurance reimbursement policies, a deficient integrity system, and inadequate oversight in managing the medical insurance fund [11, 12].

### Financing strategies of hospitals in China

Like any investor owned and nonprofit firms, hospitals finance their investment and operations through a combination of internally accumulated net incomes and external funds, which include direct fiance, mezzanine finance and indirect finance. In China, only a few hospitals are affiliated with publicly listed entities, making it impossible for them to raise funds directly by issuing equities and bonds on stock exchanges, such as through an IPO. Other means of direct finance such as taking financial lease and receiving investment from private equity firms, are not widely available for private hospitals, which are often in need of financing opportunities the most [13]. Emerging innovative financing instruments for private hospitals such as public-private-partnership (PPP) and asset-backed-securities, for which the impact on hospital sector has not been empirically tested for their impact in hospital sector [14].

Lacking sufficient means of direct finance, indirect debt financing plays a critical role in the hospital sector of China. The accessibility of bank loans is likely to vary between public and private hospitals. Without government endorsement, a long payback period and uncertain operating results could make private hospitals unappealing to the risk management departments of state-owned commercial banks. Private hospitals may have to bear higher interest-rate for the loan from the same bank than their public counterparts. Alternatively, debt financing is available for private hospitals from some private entities with a hefty interest rate. Similar patterns were observed in the United States, where a 1.6 percent difference in the cost of debt between FP hospitals and NFP hospitals was found during the period of 1972-83 [15]. A hospital’s debt financing decisions can be outlined using the following two equations based on fundamental accounting principles:

1 $$\begin{aligned} \text {Total Assets} = \text {Total Liabilities} + \text {Equity} \end{aligned}$$

2 $$\begin{aligned} \text {Total Liabilities} & = \text {Current Liabilities} + \text {Non-current Liabilities} \end{aligned}$$

Assets include fixed assets such as equipment, devices, and the hospital building, as well as current assets providing liquidity for daily operations. These assets need to be financed either through borrowing (liabilities) or accumulated net wealth (equity). The nature of borrowing can be either short-term (current liabilities) or long-term (non-current liabilities). The combination of debt and equity is the manifestation of a hospital’s capital structure.

Since most hospitals in China are nonprofit organizations, “equity” essentially represent “net assets” in the absence of investors’ contributions. The distinctions between NFP and FP hospitals in financing and operating decision-making process are blurred. More specifically, there are four distinctions in financing between FP and nonprofit organizations, which include: 1) having no owner, 2) donors influencing how the institution employs its assets, 3) immunity from involuntary bankruptcy and 4) the ability to sell tax-exempt bonds [16]. None of these distinctions effectively differentiate between the FP, NFP and public hospitals in China. For FP and nonprofit hospitals (i.e., NFP and public hospitals), the underlying assumptions for the borrowing behavior are similar. Thus, one empirical model based on capital structure theories can be used for FP hospitals and other nonprofit hospitals, as long as we account for the differences between the two groups in terms of the effect of earnings (e.g., ROA, return on assets) on capital structure.

### Related literature

#### Static trade-off theory and pecking order theory

As one of the two predominate theories of capital structure (for a comprehensive review on theories of capital structure see [17]), the static trade-off theory emerged from a series of studies initiated by Modigliani [18]. According to this theory, managers determine the desired level of debt by evaluating the benefits and the costs of borrowing [19]. One of the benefits of using debt is the tax shield allows for-profit firms to deduct interest payments from income. However, this effect is further complicated with the presence of non-debt tax shield and personal taxes, and may also affect nonprofit firms [20, 21]. Another benefit is that debt financing helps mitigate the inappropriate use of free cash flow resulting from the principal-agent problem [22]. Free cash follow can be better controlled by the principal, limiting the amount of cash available to the agent. On the flip side, borrowing that demands regular and inflexible cash outflows may cause financial distress for a firm, especially when leverage is excessive. The potential financial distress associated with the rigid payment schedules imposed by debt financing represents a primary cost [23].

How to balance the benefits and costs of debt is also related to the relationship between risk-bearing and decision-making process, which can either be combined or separated. When the risk-bearing and decision-making process are separated, managers do not bear the wealth effects of their decisions [24]. In the context of hospital industry in China, the combination of decision management, decision control and risk-bearing is expected in small FP hospitals (e.g., private level 1 hospital), while the separation of these three functions are the norm in larger private hospitals (e.g., both FP and NFP hospital level 2 and above) and public hospitals. Additionally, financing cost is also an important factor. A firm may have an optimum leverage target that is unattainable due to factors like the cost of using debt instruments. For instance, the cost of using debt may be higher for private hospitals in China, preventing them from obtaining sufficient leverage. In short, according to the static trade-off theory, leverage is expected to be greater in a firm with higher earnings, holding all other factors constant.

The pecking order theory is an alternative theory to explain how firms determine capital structure among internal revenues, debt and equity [25]. Due to information asymmetry, firms prefer to finance with internally generated funds rather than seeking external funds. This is because the financial market requires an additional risk premium from firms due to its limited information about the assets and revenues of the firms [26]. In other words, asymmetric information costs make external financing more expensivie for firms. To reduce the undesired default risk, managers may choose to use debt instruments only for residual financing needs that cannot be met by internal cash flows. In this circumstance, the pecking order theory implies a negative relationship between an earnings variable, such as ROA and leverage.

#### Empirical research on hospital and other nonprofit organization

A model to investigate the capital structure of hospitals was developed by building upon the mathematical presentation of the earlier static trade-off theory [27, 28]. The empirical test was conducted on a mixture of 1,407 FP, NFP, and public hospitals, using a linear regression model with long-term debt to assets as the outcome variable. The benefit of debt financing is captured by tax shield benefit, while the costs, which represent the risk of potential financial distress is measured using volatility of annual earnings. The signs on the tax shield benefit and volatility of earnings are positive and negative respectively, providing support evidence to the static trade-off hypothesis.

Hypotheses from both the static trade-off theory and pecking order theory were tested within a single linear regression model, using a data set of 181 NFP hospitals from 1986 to 1989 [29]. The dependent variable for leverage is total debt-to-asset ratio rather than the long-term long-term debt to assets ratio. The independent variables include leverage lagged three years, mean earnings, asset growth, volatility of earnings, the ratio of fixed assets to total assets, the logarithm of total assets, and the percentage of Medicare patients. The results show a negative coefficient on earnings and an insignificant coefficient on volatility of earnings, both of which are in favor of the pecking order theory.

A similar linear model to Bacon’s was proposed with a few changes to the independent variables [16]. The hypotheses were tested for the two competing capital structure theories using a much larger dataset that includes non-profit hospitals, universities, and human service agencies. In this study, asset growth and earnings were replaced by ROA, and the lagged leverage term was dropped to establish a fixed-effects model. The findings suggest that managers from nonprofit hospitals use static trade-off theory to guide their decisions regarding debt in operation activities.

Debt levels in FP and NFP hospitals were compared using a model with four factors: profitability, risk, growth, and size [30]. The researchers found that the NFP hospitals are associated with much lower total debt-to assets ratio than their FP counterparts. Moreover, the sensitivity of debt levels to these four factors differs significantly between FP and NFP hospitals. Using data from US nationwide short-term general acute hospitals spanning the years 2000 to 2012, a study observed that NFP hospitals exhibited less flexibility in their capital structure compared to FP hospitals, and the study identified significant effects of state-level corporate tax rates and personal income tax rates on hospital capital structure [31].

There are a few studies that have employed different model specifications to investigate non-profit organizations other than hospital. For example, in a study, a pooled cross-section Heckman selection model was implemented on a panel dataset of arts and culture organizations [4]. The first stage is to estimate the probability that an organization issue debt, while the second stage controls for selection bias and explains the long term financial debt-to-asset ratio. The primary variables of interest are the diversification of revenue which is a measure of risk, and the percentage of surplus to total revenue to capture the “profitability” of an organization. One important finding is that a more diversified revenue structure has a significant impact on an organization’s probability of issuing debt but does not precisely determine the exact financial debt ratio. Furthermore, “profitability” has no significant effect on the long-term financial debt-to-asset ratio. These findings align with predictions from the pecking order theory. Another study took advantage of a sample of over 90,000 non-profit organizations and employed a fixed-effects method to estimate a similar model, with total debt-to-asset ratio as the dependent variable [4, 5]. The fixed-effects estimators appear to support the pecking order theory.

The existing literature on hospital borrowing predominantly focuses on the United States, with limited attention given to hospitals in low- and middle-income countries, as indicated by the research reviewed above. Adequate capital is crucial for hospitals to achieve their mission of bolstering health systems and cultivating healthy communities. Given the unique characteristics of each country’s hospital sector, a nuanced examination of hospital capital structure is warranted. In the context of China, it is particularly pertinent to explore the relationships between hospital capital structure and variables such as public ownership, hospital tier, and regional market share.

Additionally, the short-term debt-to-asset ratio is frequently neglected in academic studies. Although the determinants of short-term and long-term debt-to-asset ratios may overlap, their impact magnitudes can differ substantially. Research that solely focuses on long-term debt or aggregates both short-term and long-term debt may compromise the richness and depth of insights generated from such analyses. In this study, we utilize a two-part model to examine the static trade-off theory and pecking order theory across short-term, long-term, and total debt-to-asset ratios. We incorporate variables relevant to the Chinese policy context, such as public ownership, hospital tier, and hospital type. Moreover, we include hospital’s regional market share by computing the Herfindahl-Hirschman Index for each hospital.

## Methods

### Data source

This analysis uses hospital level data from the annual statistical reports on hospitals in 2013 and 2014, which are sourced from the Health Statistical Information Report System collected by a Provincial Health Commission in the central region of China. We have limited the sample to general hospitals, Traditional Chinese Medicine (TCM) hospitals, and specialized hospitals, as other health organizations such as community health centers and township clinics may exhibit significant variations by nature. Therefore, these organizations have been excluded from our sample. Additionally, we further excluded a few hospitals with extremely high leverage ratios (higher than 1.0), indicating a potential data-entry error or a going-concern problem. To be included in our sample, hospitals were required to provide complete financial information relevant to our dependent and independent variables. This resulted in a final dataset of 1,599 observations from 909 hospitals: 959 observations from public hospitals, 208 from private NFP hospitals, and 432 from private FP hospitals.

### Measures of variables

The dependent variables are the hospital’s total debt-to-asset ratio (DA), short-term debt-to-asset ratio (SDA), and long-term debt-to-asset ratio (LDA). The values of debt and assets are based on the hospital’s self-reported book value.

The crucial independent variable is a measure of hospital earnings or profitability. As shown in Eq. 3, the variable ROA is not the same as the conventional net income in the numerator. The reason of this deviation is that costs related to depreciation, interests and taxes are not collected in the data set. In this case, ROA indicates how effectively a hospital utilizes its assets to generate net revenue, providing valuable insights to stakeholders about operational efficiency. A higher ROA reflects stronger asset utilization, which is particularly beneficial for management in demonstrating financial sustainability and operational performance.

3 $$\begin{aligned} \text {ROA} = \text { Net revenue from operations/ Total Assets} \end{aligned}$$

Another variable of interest in this analysis is the revenue stream which is captured by the revenue diversification index (DI). The DI is computed as in [4],

4 $$\begin{aligned} \text {Diversification Index}\,(\textrm{DI})=\frac{1-\sum \nolimits _{i=1}^{3} R_{i}^{2}}{0.667} \end{aligned}$$

where $R_i$ represents the ratio of revenue generated by providing medical services (PCTMED), receiving government subsidies (PCTGOV), and engaging in other activities such as revenues from development and education projects. A higher index implies a more evenly distributed revenue stream and lower financial risk for a hospital.

We account for hospital characteristics using a set of binary variables: PUBLIC, indicating public ownership (equal to 1 if the hospital is publicly owned and 0 otherwise); PROFIT, representing for-profit status (equal to 1 if the hospital is for-profit and 0 otherwise); GENERAL, identifying general hospitals (equal to 1 if the hospital is a general hospital and 0 otherwise); LVL1, for level 1 hospitals (equal to 1 if the hospital is a level 1 hospital and 0 otherwise); LVL2, for level 2 hospitals (equal to 1 if the hospital is a level 2 hospital and 0 otherwise); and LVL3, for level 3 hospitals (equal to 1 if the hospital is a level 3 hospital and 0 otherwise), with unrated hospitals serving as the reference group. Public ownership and higher-level status may indicate lower financial risk, including reduced bankruptcy risk.

Since smaller hospitals are typically associated with higher financial risk, we control for hospital size using the logarithm of total assets in 2013 values (LNASSET). Holding all else constant, larger hospitals are assumed to have better access to the capital market, which may result in higher leverage than smaller hospitals. To determine whether the debt is used to fiance durable assets such as medical devices and equipment, we include the ratio of the hospital’s fixed assets to total assets (FIXPCT). The financial strength of a hospital is captured by the logarithm of total revenue (LNREV). An increase in total revenue may raise the short term debt-to-asset ratio while reducing the long term debt-to-asset ratio [32].

Market-share is a hospital’s total revenue share in the market area. The market area is defined based on the Codes for Administrative Divisions specified to the county/municipal district level. The Herfindahl-Hirschman Index (HHI) is computed as follows:

5 $$\begin{aligned} \text {HHI}_{\text {i}}=\sum \limits _{i=1}^{n}\left( \text {S}_{\text {ij}}\right) ^{2} \end{aligned}$$

where HHI is the Herfindahl-Hirschman Index for area i rangeing from 1/n to 1, n is the total number of hospitals in the area, and $S_{ij}$ represents market-share of hospital j in area i.

It is not unreasonable to assume that the hospital’s behavior regarding debt financing is associated with agency relationships in hospital management [22, 24]. While agency cost is typically depicted as a conflict of interest between management and owner, it can be approximated by the percentage of total expenses that are spent on employee compensation (COMPPCT). Organizations providing higher employee compensations are more likely to finance with debt [33].

### Statistical analyses

In this subsection, we discuss modeling strategies for the debt-to-asset ratios. For all three debt-to-asset ratios, there is a considerable number of observations where the ratio is zero. Specifically, the observed number of zero ratios is 190, 223, and 810 (out of 1599 observations) for the total, short-term and long-term debt-to-asset ratio respectively. Since the relevant methodological issues are practically identical for these three ratios we will generalize the discussion to debt-to assets ratios as a whole. These zero observations may occur for three main reasons: Firstly, a hospital may prefer financing by using internal funds and there is sufficient funds to meet its needs. Secondly, a hospital may be a potential applicant of bank loan and it is considered unqualified (e.g., rejected by the the bank’s risk management) to get the loan. Thirdly, a hospital may have retired its debt before reporting the financial data, given that such data is only collected once a year.

A single equation model is likely to produce biased estimates, considering the mass points at zero. There are two common approaches to account for the mass zeros: the Heckman selection model and the two-part model [34]. From a theoretical perspective, the choice between a Heckman selection and a two-part model depends on whether we wish to model potential or actual outcomes [35]. For instance, the selection model is applicable in contexts like estimating wage equations. In such cases, we are not only trying to estimate the effect of a covariate, such as schooling on wages among these who actually worked and their wages were observed. More importantly, we are interested in modeling the potential wages individuals could earn if they were to work. It does not apply to the debt-to-asset ratios. For hospitals with observed zero debt, there is no latent positive expected debt-to-asset ratio to be modeled.This study uses a two-part model, commonly applied in financial research, to analyze hospital debt decisions. The first part estimates the likelihood of a hospital having any debt, while the second examines the amount of debt for those with positive debt levels [36].

Firstly, using pooled cross-sectional data from 2013 and 2014, the probability that a hospital has positive debt-to-asset ratios is modeled as in [37]

6 $$\begin{aligned} \phi (y>0) = \text {Pr}(y>0 \mid \textbf{x}_{\varvec{1}})=N(\textbf{x}_{\varvec{1}} \varvec{\beta }). \end{aligned}$$

The outcome *y* is one of the three debt-to-asset ratios. The vector $\textbf{x}_{\varvec{1}}$ includes all explanatory variables listed in Table 2, except the level of hospital, LVL. After controlling for other independent variables such as PUBLIC, PROFIT and LNASSET, the hospital’s level may have little effect on the probability of positive debt-to-asset ratios. However, LVL is critical to determine the positive debt-to-asset ratios. This is because higher level hospitals require more capital investments in equipment and devices for inpatient services, while lower level hospitals are focus in primary care which demands less capital. The vector $\varvec{\beta }$ represents the corresponding parameters to be estimated, and *N* denotes the cumulative distribution function of an independent, identically and normally distributed error term.

For the observations with positive debt-to-asset ratios, the model can be written as

7 $$\begin{aligned} \phi (y \mid y>0, \textbf{x})=f(\textbf{x} \varvec{\gamma }) \end{aligned}$$

where $\textbf{x}$ is a vector contains all independent variables listed in Table 2, as well as an interaction term between PROFIT and ROA. The interaction term allows us to account for the different financing behavior between non-profit and FP hospitals. For example, FP hospitals may employ higher leverage than non-profit hospitals given the same level of ROA to maximize profits. The corresponding vector of parameters to be estimated is denoted as $\varvec{\gamma }$, and *f* is the density function for $y|y>0$. Taken together, Eqs. (5) and (6), the likelihood contribution for an observation is represented as

8 $$\begin{aligned} \phi (y)=\{1-N(\textbf{x}_{\varvec{1}} \varvec{\beta })\}^{i(i=0)} \times \{N(\textbf{x}_{\varvec{1}}\varvec{\beta }) f(\textbf{x} \varvec{(\gamma )}\}^{i(y>0)} \end{aligned}$$

where i($\varvec\cdot$) denotes the indicator function. So the log-likelihood contribution is

9 $$\begin{aligned} \ln \{\phi (y)\} & = i(i=0) \ln \{1-N(\textbf{x}_{\varvec{1}} \varvec{\beta })\}\nonumber \\ & \quad +i(i>0)[\ln \{F(\textbf{x}_{\varvec{1}} \varvec{\delta })\}+\ln \{f(\textbf{x} \varvec{\gamma })\}] \end{aligned}$$

Because the $\varvec{\beta }$ and $\varvec{\gamma }$ parameters have the additively separable feature in the log-likelihood contribution for each observation, the models for the zeros and the positive debt-to-asset ratios can be estimated separately. Firstly, we model $\text {Pr}(y>0 \mid \textbf{x}_{\varvec{1}})$ as a probit. In the second part, the positives values $E(y \mid y>0, \textbf{x})$ can be modeled by a linear regression using ordinary least squares (OLS) or a generalized lineal model (GLM). Given the three outcome variables of debt-to-asset rations are defined on the unit internal of [0,1], the shortcomings of OLS models for fractional data parallel the shortcomings of linear probability models for binary data. A common alternative to OLS on the second part is fractional regression model (FRM), which is fitted similarly to a GLM [36, 38, 39].

## Results

### Descriptive results

The descriptive statistics for each of the variables in the analysis are listed in and Table 2.

**Table 2:** Descriptive statistics of variables

Variable	Definition	Mean	Standard deviation
Dependent:			
DA	Total debt-to-asset ratio	0.431	0.292
SDA	Short-term debt-to-asset ratio	0.312	0.243
LDA	Long-term debt-to-asset ratio	0.119	0.185
Independent:			
ROA	Net revenue from operations/Total Assets	0.368	0.714
DI	Revenue diversification index	0.153	0.214
PCTGOV	Percent revenue from government subsidies	0.045	0.098
PCTMED	Percent revenue from medical services	0.931	0.122
PUBLIC	Dummy variable; value is one when a hospital is public owned, zero otherwise.	0.600	0.490
PROFIT	Dummy variable; value is one when a hospital is for profit, zero otherwise.	0.279	0.449
GENERAL	Dummy variable; value is one when a hospital is general hospital, zero otherwise.	0.599	0.491
LVL1	Dummy variable; value is one when a hospital is level 1 hospital, zero otherwise.	0.243	0.429
LVL2	Dummy variable; value is one when a hospital is level 2 hospital, zero otherwise.	0.323	0.468
LVL3	Dummy variable; value is one when a hospital is level 3 hospital, zero otherwise.	0.079	0.270
LNASSET	Log of total assets	9.671	2.084
FIXPCT	Ratio of fixed assets to total assets	0.485	0.235
LNREV	Log of total revenue	9.572	2.013
HHI	Herfindahl index for the area	0.471	0.140
COMPPCT	Ratio of compensation to total expenses	0.353	0.143

Note: There are 1599 observations for 909 hospitals in year 2013 and 2014

### Regression results

There are three outcome variables in the regression models: the first dependent variable is total debt-to-asset ratios (DA), the second is short-term debt-to-asset ratios (SDA), and the third is long-term debt-to-asset ratios (LDA). Table 3 presents coefficients and standard errors from the first part probit analysis. Tables 4 and 5 display the estimation results from the second part analysis using OLS and FRM, respectively. The pseudo $R^2$ and $R^2$ values indicate that our two-part model accounts for a considerable portion of the variation in dependent variables. Several coefficients attain statistical significance at the ten percent level or better based on a two-tailed t-test.

**Table 3:** The first part of capital structure regressions-probit

	DA	SDA	LDA
ROA	−0.200**	−0.223***	0.033
	(0.085)	(0.087)	(0.098)
DI	0.199	0.190	−0.494
	(0.653)	(0.554)	(0.497)
PCTGOV	2.100**	1.222	−0.264
	(1.075)	(0.925)	(0.916)
PCTMED	1.876*	2.237**	−0.620
	(1.138)	(1.056)	(1.110)
PUBLIC	−0.023	0.228	0.359**
	(0.183)	(0.177)	(0.154)
PROFIT	−0.221	−0.117	−0.035
	(0.175)	(0.164)	(0.150)
GENERAL	−0.055	−0.037	−0.056
	(0.128)	(0.121)	(0.093)
LNASSET	0.100	0.128*	0.304***
	(0.071)	(0.071)	(0.068)
FIXPCT	−0.989***	−1.213***	0.123
	(0.262)	(0.256)	(0.199)
LNREV	0.269***	0.234***	0.031
	(0.067)	(0.067)	(0.065)
HHI	0.667	0.441	1.252***
	(0.517)	(0.489)	(0.333)
COMPPCT	0.948***	0.893**	0.536*
	(0.376)	(0.358)	(0.321)
Constant	−3.778***	−4.034***	−3.212***
Pseudo $R^2$	0.266	0.279	0.220
N	1599	1599	1599

Notes: Heteroscedastic-robust standard errors clustered at hospital level in parentheses. *Significant at 10%, **significant at 5%, ***significant at 1%. Pseudo $R^2$ is obtained using the McFadden’s approach

**Table 4:** The second part of capital structure regressions- OLS

	DA	SDA	LDA
ROA	0.013	−0.005	0.002
	(0.023)	(0.022)	(0.024)
PROFIT	0.013	−0.021	0.013
	(0.036)	(0.033)	(0.059)
PROFIT*ROA	−0.000	0.001	0.067*
	(0.029)	(0.030)	(0.038)
DI	−0.252**	−0.212*	0.051
	(0.120)	(0.122)	(0.092)
PCTGOV	−0.439**	−0.244	−0.297
	(0.179)	(0.151)	(0.199)
PCTMED	−0.495**	−0.358	−0.119
	(0.231)	(0.227)	(0.189)
PUBLIC	−0.007	−0.01	−0.066
	(0.033)	(0.032)	(0.046)
GENERAL	0.008	−0.018	0.037**
	(0.019)	(0.016)	(0.019)
1.LVL	0.010	−0.008	0.001
	(0.028)	(0.025)	(0.040)
2.LVL	0.114***	0.031	−0.010
	(0.037)	(0.030)	(0.043)
3.LVL	0.102**	0.042	−0.030
	(0.050)	(0.039)	(0.055)
LNASSET	0.000	−0.047***	0.036*
	(0.014)	(0.013)	(0.019)
FIXPCT	−0.166***	−0.144***	−0.062
	(0.042)	(0.036)	(0.045)
LNREV	0.003	0.039***	−0.036**
	(0.015)	(0.013)	(0.018)
HHI	−0.053	−0.115**	−0.014
	(0.062)	(0.055)	(0.064)
COMPPCT	−0.061	−0.118**	0.035
	(0.070)	(0.060)	(0.082)
Constant	1.031***	0.993***	0.408**
	(0.253)	(0.236)	(0.193)
$R^2$	0.093	0.075	0.086
N	1409	1376	789

Notes: Heteroscedastic-robust standard errors clustered at hospital level in parentheses. *Significant at 10%, **significant at 5%, ***significant at 1%

**Table 5:** The second part of capital structure regressions- FRM

	DA	SDA	LDA
ROA	0.012	−0.005	0.000
	(0.023)	(0.021)	(0.026)
PROFIT	0.012	−0.023	0.007
	(0.036)	(0.033)	(0.052)
PROFIT*ROA	−0.000	0.001	0.054
	(0.029)	(0.029)	(0.035)
DI	−0.256**	−0.215*	0.058
	(0.124)	(0.120)	(0.096)
PCTGOV	−0.449**	−0.247	−0.288
	(0.185)	(0.151)	(0.192)
PCTMED	−0.502**	−0.354	−0.088
	(0.243)	(0.221)	(0.183)
PUBLIC	−0.007	−0.016	−0.066
	(0.033)	(0.032)	(0.043)
GENERAL	0.008	−0.020	0.037**
	(0.019)	(0.016)	(0.018)
1.LVL	0.011	−0.009	0.006
	(0.028)	(0.025)	(0.037)
2.LVL	0.113***	0.030	−0.008
	(0.037)	(0.030)	(0.041)
3.LVL	0.102**	0.038	−0.025
	(0.050)	(0.039)	(0.052)
LNASSET	0.000	−0.047***	0.033*
	(0.014)	(0.013)	(0.017)
FIXPCT	−0.168***	−0.143***	−0.064
	(0.041)	(0.036)	(0.044)
LNREV	0.000	0.039***	−0.033**
	(0.014)	(0.013)	(0.016)
HHI	−0.053	−0.113	−0.039
	(0.065)	(0.055)	(0.065)
COMPPCT	−0.061	−0.119**	0.031
	(0.070)	(0.061)	(0.076)
Pseudo $R^2$	0.058	0.035	0.046
N	1409	1376	789

Notes: Only the average marginal effects are reported. Delta-method standard errors clustered at hospital level in parentheses. *Significant at 10%, **significant at 5%, ***significant at 1%. Pseudo is obtained using the McFadden’s approach

The return-on-assets (ROA) clearly has a negative impact on the probability that a hospital take short-term debt, but has no significant impact on the probability of obtaining long-term debt. This finding suggests that hospitals’ decisions regarding short-term debt align more closely with the pecking order theory, indicating a preference for utilizing internal funding first. The diversification of revenue stream does not affect the probability of hospital assuming debt. Moreover, neither PROFIT nor GENERAL is statistically significant at predicting the probability of having positive debt-to-asset ratios. The positive sighs associated with PUBLIC on SDA and LDA are consistent with the view that public hospitals have better chance to get bank loans. It seems that public hospitals really have an edge over private hospitals for obtaining long-term loans. This phenomenon may be due to banks viewing the approval of loans, especially long-term ones, to private hospitals as associated with higher risk. While the local government may step in if a public hospital faces financial difficulties, this may not be the case for private hospitals.

The parameter estimates on explanatory variables represents hospital bankruptcy risk have the anticipated sighs. The coefficients on log of assets (LNASSET)and log of revenues (LNREV) are both positive and statistically significant. Hospitals with more assets and more revenue are also more likely to have positive debt-to-asset ratios. Holding all other variables constant, hospitals in a more concentrated market are more likely to take long term debt but not short term debt. Hospitals operating in a more concentrated market with heightened competition may have a greater need for long-term projects and expansion to gain a competitive edge. Additionally, more concentrated markets may allow hospitals to benefit from economies of scale, in which they use long-term debt to finance the acquisition of advanced medical equipment, the construction of larger facilities that can be cost-effective in the long run.

The fixed-assets ratio (FIXPCT) has a significant negative effect on the probability of having short-term debt and no significant effect on having long-term debt. This finding may be attributed to the correlation between a higher fixed-assets ratio and better ability to generate operating revenues. Hospitals with a higher fixed-assets ratio may generate sufficient cash flows from daily operations and are less likely to need short-term debt financing. The ratio of compensation to total expenses (COMPPCT) shows a systematic positive relationship with the probability of having positive debt-to-asset ratios.

The coefficients from the OLS regression models in the second part are presented in Table 4, and these results closely resemble the average marginal effects obtained from the FRM regression models shown in Table 5. In contrast to the first-part probit analysis, ROA is not statistically significant in determining the magnitude of debt-to-asset ratios. However, the coefficient on PROFIT*ROA is statistically significant when long-term debt-to-asset is the outcome variable. This suggests that private FP hospitals may more actively evaluate the costs of debt against the benefits (e.g., ROA) when assuming long-term debt, aligning with the predictions of the trade-off theory. Additionally, revenue diversification (DI) negatively influences the hospital’s short-term debt-to-asset ratio. A hospital with a more diversified revenue stream may indicates a more stable revenue structure, in turn, requires lower leverage in the short-term to meet operation needs.

Holding other factors constant, none of the hospital characteristics such as PROFIT, PUBLIC, and LVL is a significant predictor of the hospital short-term or long-term debt-to-asset ratios. The only exception is GENERAL, for which being a general hospital is associated with higher long-term debt-to-asset ratios. Hospitals in more concentrated markets, on average, are associated with lower short-term debt ratios. It is worth noting that, higher level hospitals (level 2 and level 3) are associated with higher total debt-to-asset ratios.

The logarithm of assets (LNASSET) and the logarithm of revenues (LNREV) exert opposite influences on determining short-term and long-term debt-to-asset ratios. Greater total assets are associated with higher long-term debt-to-asset ratios and lower short-term debt-to-asset ratios. Conversely, higher total revenue is linked to higher short-term debt-to-asset ratios and lower long-term debt-to-asset ratios.

The ratio of fixed assets to total assets (FIXPCT) has a negative effect on hospital short-term dent-to-assets ratios. A similar pattern is observed in the first part and the logic of the coefficient sigh applies here as well. The negative sigh of estimate on COMPPCT from the SDA regression is unanticipated. One possible explanation is that higher compensation is a indicator of higher skilled pool of physicians. In the sub-sample of hospitals having positive short-term debt-to-asset ratios, more skilled medical staff are capable of generate more stable cash flow from daily operation. In turn, the leverage of short-term debt are lower for hospitals pay a higher percentage of compensation relative to total expenses.

### Not-for-profit hospitals versus for-profit hospitals

Results from Tables 4 and 5 indicate that for-profit (FP) hospitals may adopt a different approach than not-for-profit (NFP) hospitals when assuming long-term debt, placing greater emphasis on ROA. To further differentiate the financial leverage decision-making processes between NFP and FP hospitals, we re-estimate the regression models by separating them into distinct sub-samples. It is worth noting that all observations from public hospitals in our dataset are NFP, and 31 percent observations from private hospitals have NFP status. We re-estimate the two-part models separately by hospital profit status, retaining all previously described variables. However, we exclude the variable PUBLIC due to its causing a perfect collinearity problem.

In Table 6, we present the regression coefficients from the first-part probit analysis, categorized by hospital profit status. The model fit was notably better for NFP hospitals than for FP hospitals. The estimates for NFP hospitals were consistent with those presented in Table 3. NFP hospitals tend to prioritize internal funding over assuming short-term debt, which aligns with the pecking order theory. However, there was no clear evidence supporting the pecking order theory for long-term debts in NFP hospitals, or for both short-term and long-term debts in FP hospitals.

**Table 6:** The first part of capital structure regressions by profit status-probit

	NFP hospitals			FP hospitals		
	DA	SDA	LDA	DA	SDA	LDA
ROA	−0.274**	−0.206*	0.014	−0.052	−0.181	0.156
	(0.135)	(0.124)	(0.135)	(0.109)	(0.114)	(0.149)
DI	0.359	0.353	−0.231	−7.624*	−6.754**	−4.418
	(0.674)	(0.596)	(0.480)	(4.012)	(3.423)	(2.783)
PCTGOV	2.103*	1.220	−1.018	−2.695	−0.449	0.514
	(1.210)	(1.055)	(0.874)	(3.694)	(3.327)	(2.585)
PCTMED	2.291**	2.566**	−1.078	−16.480*	−13.371*	−8.275
	(1.154)	(1.156)	(1.011)	(9.283)	(7.463)	(5.972)
GENEARL	−0.179	−0.243	−0.202*	0.083	0.200	0.356**
	(0.204)	(0.193)	(0.113)	(0.182)	(0.177)	(0.172)
LNASSET	0.080	0.172*	0.226**	0.109	0.088	0.271***
	(0.101)	(0.099)	(0.090)	(0.098)	(0.097)	(0.098)
FIXPCT	−1.241***	−1.529***	0.261	−0.781**	−0.875**	−0.139
	(0.393)	(0.389)	(0.250)	(0.371)	(0.358)	(0.339)
LNREV	0.289***	0.212**	0.190**	0.208**	0.225**	−0.223**
	(0.096)	(0.096)	(0.094)	(0.094)	(0.094)	(0.090)
HHI	0.344	0.001	0.945**	1.008	0.555	0.711
	(0.774)	(0.698)	(0.414)	(0.667)	(0.658)	(0.627)
COMPPCT	1.255**	1.232**	0.056	0.619	0.492	0.927*
	(0.573)	(0.541)	(0.434)	(0.507)	(0.489)	(0.505)
Constant	−3.857***	−4.000***	−3.298***	14.533	11.667	6.398
	(1.397)	(1.370)	(1.121)	(9.300)	(7.483)	(6.045)
Pseudo $R^2$	0.345	0.353	0.241	0.099	0.115	0.068
N	1153	1153	1153	439	439	439

Notes: Heteroscedastic-robust standard errors clustered at hospital level in parentheses. *Significant at 10%, **significant at 5%, ***significant at 1%. Pseudo $R^2$ is obtained using the McFadden’s approach

Table 7 presents the results from the second-part OLS analysis. Consistent with the findings in Table 4, we observe that a higher ROA is associated with a higher long-term-to-debt ratio in FP hospitals, supporting the trade-off theory. However, similar findings were not observed in NFP hospitals. Additionally, the impact of market share, as observed in Table 4, appears to be constrained in FP hospitals. Specifically, in FP hospitals operating in more concentrated markets, a higher short-term-to-debt ratio is observed.

**Table 7:** The second part of capital structure regressions by profit status-OLS

	NFP hospitals			FP hospitals		
	DA	SDA	LDA	DA	SDA	LDA
ROA	0.022	0.006	−0.000	0.012	−0.014	0.076**
	(0.025)	(0.024)	(0.031)	(0.025)	(0.026)	(0.036)
DI	−0.265**	−0.247**	0.068	−0.744	0.250	−2.345***
	(0.120)	(0.123)	(0.098)	(0.528)	(0.718)	(0.650)
PCTGOV	−0.500***	−0.240	−0.290	0.113	−0.060	−1.150***
	(0.189)	(0.163)	(0.191)	(0.442)	(0.470)	(0.336)
PCTMED	−0.519**	−0.390*	−0.000	−1.516	0.482	−5.171***
	(0.236)	(0.233)	(0.177)	(0.926)	(1.390)	(1.132)
GENEARL	−0.010	−0.022	0.045**	0.059	−0.008	0.033
	(0.021)	(0.017)	(0.019)	(0.044)	(0.040)	(0.065)
1.LVL	0.010	−0.014	−0.037	−0.012	−0.009	0.034
	(0.034)	(0.031)	(0.050)	(0.050)	(0.043)	(0.085)
2.LVL	0.107***	0.026	−0.030	−0.090	−0.061	−0.087
	(0.041)	(0.033)	(0.053)	(0.145)	(0.078)	(0.121)
3.LVL	0.082	0.033	−0.029	NA	NA	NA
	(0.056)	(0.045)	(0.066)			
LNASSET	0.008	−0.036**	0.045*	−0.009	−0.057***	0.042
	(0.017)	(0.018)	(0.024)	(0.025)	(0.020)	(0.036)
FIXPCT	−0.141***	−0.115***	−0.073	−0.183**	−0.195**	0.046
	(0.045)	(0.039)	(0.048)	(0.091)	(0.080)	(0.137)
LNREV	0.001	0.031	−0.055**	−0.006	0.039**	−0.021
	(0.018)	(0.019)	(0.024)	(0.024)	(0.017)	(0.035)
HHI	0.009	−0.064	0.064	−0.299**	−0.284**	−0.094
	(0.071)	(0.061)	(0.067)	(0.140)	(0.123)	(0.284)
COMPPCT	−0.025	−0.074	0.110	−0.112	−0.166	−0.043
	(0.087)	(0.071)	(0.108)	(0.125)	(0.110)	(0.140)
Constant	0.961***	0.947***	0.298*	2.338**	0.328	5.300***
	(0.259)	(0.250)	(0.177)	(0.998)	(1.407)	(1.117)
$R^2$	0.130	0.065	0.061	0.076	0.120	0.112
N	1061	1042	668	348	334	121

Notes: Heteroscedastic-robust standard errors clustered at hospital level in parentheses. There was no Level 3 for-profit hospital in the sample. *Significant at 10%, **significant at 5%, ***significant at 1%. Pseudo $R^2$ is obtained using the McFadden’s approach

## Discussion

This study developed two major themes. First, we examined the effect of ownership, hospital type, revenue stream, market share, and other hospital characteristics on hospital leverage ratios. We employed a two-part model to investigate the relationship between these hospital characteristics and the probability of having positive debt-to-asset ratios. We then explored to what extent these factors determine debt-to-asset ratios within indebted hospitals.

We found that being a private FP hospital and being a non-general hospital do not significantly decrease the hospital’s probability of taking on short-term debt. A private FP hospital with a less diversified revenue stream is associated with a higher probability of assuming short-term debt, potentially to cover operational needs. Additionally, private ownership significantly decreases the probability of a hospital assuming long-term debt.

The story is different when we look at the factors that determine the debt-to-asset ratios. Holding all else constant, higher-level hospitals are generally more indebted than lower-level hospitals in terms of total debt-to-asset ratio. General hospitals have higher long-term debt-to-asset ratios compared to specialized hospitals, at least for NFP hospitals. A more diversified revenue stream is associated with significantly lower short-term debt-to-asset ratios among FP hospitals. In contrast, NFP hospitals in a more concentrated market are more likely to assume long-term loans.

Second, we examined the capital structure decisions of hospitals, focusing on two corporate finance theories: the pecking order theory and the static trade-off theory. The results of the empirical analysis indicate that both public and private NFP hospitals display preferences consistent with the pecking order theory for short-term debt levels, preferring internal funding over external borrowing. Additionally, there is evidence that private FP hospitals consider the trade-off between the benefits and costs of borrowing when determining long-term debt levels. The results suggest that private hospitals face significant difficulties in securing long-term loans compared to public hospitals. However, those that do obtain long-term financing have debt levels similar to public hospitals. This indicates that private hospitals may face unequal access to loans, possibly due to factors like relationships with banks.

Similar to earlier studies conducted in the United States, where the type of hospital—investor-owned versus not-for-profit—is a significant factor influencing hospitals’ capital decision-making, another important consideration in China is the distinction between public and private hospitals [40, 41]. This distinction is particularly relevant given the predominant role of public hospitals in delivering care. A recent study using data from hospitals in Portugal found that both public and private hospitals rely on long-term loans during financial stress, with public hospitals being more dependent on short-term loans [42]. This finding contrasts with our results, which suggest that private hospitals in China may struggle to secure long-term loans, while public hospitals are more inclined to use internal funding rather than short-term loans. These differences highlight the importance of considering the unique characteristics of a country’s healthcare system and capital market when studying this topic. More research is needed to deepen our understanding, particularly in low- and middle-income countries, where little investigation has been conducted on this subject.

From an empirical perspective, our findings contribute to the literature on healthcare finance by highlighting how institutional constraints and ownership types shape capital structure decisions in emerging markets. The results underscore the need to account for revenue diversification and financing accessibility when modeling hospital behavior under different institutional and regulatory environments. From a managerial standpoint, hospital administrators, particularly those in private FP institutions, should recognize the importance of diversifying revenue streams and improving financial reporting transparency to strengthen their creditworthiness and ability to access long-term debt. Hospital administrators may proactively seek partnerships with investors and explore blended financing models that combine private capital with public support. These strategies can enhance financial resilience and enable long-term planning and service expansion.

There are several limitations to our study. One notable limitation of this study is our inability to differentiate between tax-exempt debt and taxable debt when analyzing the capital structure of FP hospitals. Tax-exempt debt can provide a significant tax shield effect. Therefore, our inability to separate these two types of debt in our analysis may have resulted in an incomplete understanding of the tax implications on borrowing decisions among FP hospitals. Future research could benefit from access to more detailed financial data that allows for this distinction, enabling a comprehensive examination of the tax shield effect on capital structure decisions among FP hospitals.

Another limitation relates to the scope of our data. Our dataset was derived from a single province in China and covers only two years. This geographic and temporal limitation may restrict the generalizability of our findings to a broader context. Hospital financing practices and capital structure determinants could vary significantly across different regions within China and evolve over time. To address this limitation, future research should aim to gather data from multiple provinces or regions within China for a more comprehensive and representative analysis. Additionally, expanding the dataset to cover a longer time frame and employing time-series models could capture the dynamic nature of hospital financing and capital structure changes over time. Future studies could also consider employing fixed effects models with panel data or quantile regression models to provide further insights on this topic, as similar studies have been conducted with firms listed on public exchanges [43].

Lastly, the healthcare system in China has undergone rapid reforms since 2009. Numerous policies, such as the zero-markup policy for prescription drugs dispensed in public hospitals, and changes in payment mechanisms from fee-for-service to capitated global budgets for outpatient care and Diagnosis-Related Groups (DRGs) for inpatient care, may have spillover effects on hospital revenues and, consequently, the health of hospital capital structures. These policies warrant examination in future research to understand their impact on hospital financing and capital structure.

## Conclusion

This study provides valuable insights into the capital structure decisions of hospitals in China, focusing on the effects of ownership, hospital type, revenue stream, and other key characteristics. We found that being a private for-profit (FP) hospital and being a non-general hospital do not significantly decrease the probability of assuming short-term debt. Private FP hospitals with less diversified revenue streams are more likely to assume short-term debt, possibly to cover operational needs. Additionally, private ownership significantly reduces the likelihood of assuming long-term debt, suggesting that private hospitals face substantial challenges in accessing long-term financing.

Long-term debt financing is crucial for addressing funding shortages in private hospitals, enabling them to maintain healthy capital structures and contribute to quality healthcare. The government can support this by facilitating access to long-term loans, providing subsidies, and exploring alternative financing channels such as private equity investment or the formation of publicly traded hospital companies. A well-functioning financing system for private hospitals can help them realize their potential, improving healthcare access, reducing overcrowding in public hospitals, and advancing the Healthy China 2030 initiative [44]. A robust private hospital system that complements public hospitals is essential for ensuring equitable and efficient healthcare delivery. Addressing disparities in loan accessibility and fostering sustainable financial practices will be key to enabling private hospitals to play a larger role in meeting healthcare demands in China.

## Data Availability

The data used in this research article are regulated by the government and require official approval from either the National Health Commission of the PRC or provincial Health Commission for access and use.

## References

[1] Xu J, Jian W, Zhu K, Kwon S, Fang H. Reforming public hospital financing in China: progress and challenges. BMJ. 2019;365:l4015. 31227512 10.1136/bmj.l4015 PMC6598707

[2] Ahn YC, Kim JM, Ham U, et al. Comparative Analysis on the Capital Structure of Superior Hospital and Bankrupt Hospital. Korea J Hosp Manag. 2006;11(4):19–36.

[3] Sun Z, Wang S, Barnes SR. Understanding congestion in China’s medical market: an incentive structure perspective. Health Policy Plan. 2016;31(3):390–403. 26185181 10.1093/heapol/czv062

[4] Yan W, Denison DV, Butler JS. Revenue structure and nonprofit borrowing. Public Financ Rev. 2009;37(1):47–67.

[5] Calabrese TD. Testing competing capital structure theories of nonprofit organizations. Public Budg Finance. 2011;31(3):119–43.

[6] Ke X, Saksena P, Holly A. The determinants of health expenditure: a country-level panel data analysis. vol. 26. issue 1-28. Geneva: World Health Organization; 2011.

[7] Jia H, Jiang H, Yu J, Zhang J, Cao P, Yu X. Total Health Expenditure and Its Driving Factors in China: A Gray Theory Analysis. Healthcare. 2021;9(2):207–224. 10.3390/healthcare9020207 PMC7918561 33673001

[8] Zhai T, Goss J, Li J. Main drivers of health expenditure growth in China: a decomposition analysis. BMC Health Serv Res. 2017;17(1):1–9. 28274228 10.1186/s12913-017-2119-1 PMC5343399

[9] Chen Z. Launch of the health-care reform plan in China. Lancet. 2009;373(9672):1322–4. 19376436 10.1016/S0140-6736(09)60753-4

[10] Liu X, Liu Y, Chen N. The Chinese experience of hospital price regulation. Health Policy Plann. 2000;15(2):157–63. 10.1093/heapol/15.2.157 10837038

[11] Zhu Y, Zhao Y, Dou L, Guo R, Gu X, Gao R, et al. The hospital management practices in Chinese county hospitals and its association with quality of care, efficiency and finance. BMC Health Serv Res. 2021;21(1):449. 33975605 10.1186/s12913-021-06472-7 PMC8111980

[12] Chen C, Liu M. Achievements and challenges of the healthcare system in China. Cureus. 2023;15(5):39030. 10.7759/cureus.39030 PMC10292030 37378106

[13] Hu Z. The Exploration on Financing Modes in Private Hospitals. China Health Standard Manag. 2016;7(24):11–3.

[14] Guo L. Research of the financing channels, problems and countermeasures of the private hospitals. Chin Hosp Manag. 2015;35(6):4–6.

[15] Sloan FA, Valvona J, Hassan M, Morrisey MA. Cost of capital to the hospital sector. J Health Econ. 1988;7(1):25–45. 10302653 10.1016/0167-6296(88)90003-3

[16] Bowman W. The uniqueness of nonprofit finance and the decision to borrow. Nonprofit Manag Leadersh. 2002;12(3):293–311.

[17] Harris M, Raviv A. The theory of capital structure. J Finance. 1991;46(1):297–355.

[18] Modigliani F, Miller MH. The cost of capital, corporation finance and the theory of investment. Am Econ Rev. 1958;48(3):261–97.

[19] Myers SC. Capital structure. J Econ Perspect. 2001;15(2):81–102.

[20] Miller MH. Debt and taxes. J Financ. 1977;32(2):261–75.

[21] DeAngelo H, Masulis RW. Optimal capital structure under corporate and personal taxation. J Financ Econ. 1980;8(1):3–29.

[22] Jensen MC. Agency costs of free cash flow, corporate finance, and takeovers. Am Econ Rev. 1986;76(2):323–9.

[23] Modigliani F, Miller MH. Corporate income taxes and the cost of capital: a correction. Am Econ Rev. 1963;53(3):433–43.

[24] Fama EF, Jensen MC. Separation of ownership and control. J Law Econ. 1983;26(2):301–25.

[25] Myers SC. Capital structure puzzle. J financ. 1984;39(3):575–592.

[26] Myers SC, Majluf NS. Corporate financing and investment decisions when firms have information that investors do not have. J Financ Econ. 1984;13(2):187–221.

[27] Wedig G, Sloan FA, Hassan M, Morrisey MA. Capital structure, ownership, and capital payment policy: The case of hospitals. J Financ. 1988;43(1):21–40.

[28] Bradley M, Jarrell GA, Kim EH. On the existence of an optimal capital structure: Theory and evidence. J Financ. 1984;39(3):857–78.

[29] Bacon PW. Do capital structure theories apply to nonprofit hospitals. J Midwest Financ Assoc. 1992;21(1):86–90.

[30] Turner J, Broom K, Elliott M, Lee JF. A comparison of capital structure: The use of debt in investor owned and not-for-profit hospitals. J Health Care Financ. 2015;41(4):1–17.

[31] Huang SS, Yang J, Carroll N. Taxes, bankruptcy costs, and capital structure in for-profit and not-for-profit hospitals. Health Serv Manag Res. 2018;31(1):21–32. 10.1177/0951484817726780 28876139

[32] Clingermayer JC, Wood BD. Disentangling patterns of state debt financing. Am Polit Sci Rev. 1995;89(1):108–20.

[33] Jegers M. “Corporate’’ governance in nonprofit organizations: A nontechnical review of the economic literature. Nonprofit Manag Leadersh. 2009;20(2):143–64.

[34] Deb P, Norton EC. Modeling health care expenditures and use. Ann Rev Public Health. 2018;39:489–505. 29328879 10.1146/annurev-publhealth-040617-013517

[35] Madden D. Sample selection versus two-part models revisited: the case of female smoking and drinking. J Health Econ. 2008;27(2):300–7. 18180064 10.1016/j.jhealeco.2007.07.001

[36] JS Ramalho J, da Silva JV. A two-part fractional regression model for the financial leverage decisions of micro, small, medium and large firms. Quant Financ. 2009;9(5):621–36.

[37] Belotti F, Deb P, Manning WG, Norton EC. twopm: Two-part models. Stata J. 2015;15(1):3–20.

[38] Wooldridge JM. Econometric analysis of cross section and panel data. Cambridge, MA: MIT Press; 2010.

[39] Papke LE, Wooldridge JM. Econometric methods for fractional response variables with an application to 401 (k) plan participation rates. J Appl Econ. 1996;11(6):619–32.

[40] Wheeler JRC, Smith DG, Rivenson HL, Reiter KL. Capital structure strategy in health care systems. J Health Care Financ. 2000;26(4):42–52. ASPEN PUBLISHERS, INC. 10845385

[41] Valvona J, Sloan FA. Hospital profitability and capital structure: a comparative analysis. Health Serv Res. 1988;23(3):343. Health Research & Educational Trust. PMC1065509 3403274

[42] Neves ME, Ferreira I, Serrasqueiro Z, Cancela B. Differences in capital structure between public and private hospitals: new evidence from a small country banking system using panel data. J Health Organ Manag. Emerald Publishing Limited. 2024;39(5):605–628.

[43] De Jong A, Verbeek M, Verwijmeren P. Firms’ debt-equity decisions when the static tradeoff theory and the pecking order theory disagree. J Bank Financ. 2011;35(5):1303–14.

[44] Tan X, Zhang Y, Shao H. Healthy China 2030, a breakthrough for improving health. Glob Health Promot. 2019;26(4):96–9. 29297762 10.1177/1757975917743533

